# The “Ecosystem as a Service (EaaS)” approach to advance clinical artificial intelligence (cAI)

**DOI:** 10.1371/journal.pdig.0000011

**Published:** 2022-02-03

**Authors:** Julian Euma Ishii-Rousseau, Shion Seino, Daniel K. Ebner, Maryam Vareth, Ming Jack Po, Leo Anthony Celi

**Affiliations:** 1 MIT Critical Data, Cambridge, Massachusetts, United States of America; 2 Department of Global Health Promotion, Tokyo Medical and Dental University, Tokyo, Japan; 3 General Incorporated Association Liaison, Tokyo, Japan; 4 National Institutes of Quantum and Radiological Science and Technology, Chiba, Japan; 5 Department of Radiation Oncology, Mayo Clinic, Rochester, Minnesota, United States of America; 6 Department of Radiology and Biomedical Imaging, University of California San Francisco, San Francisco, California, United States of America; 7 Berkeley Institute for Data Science, Division of Computing, Data Science, and Society, University of California Berkeley, Berkeley, California, United States of America; 8 Data Science Institute, Lawrence Livermore National Laboratory, United States of America; 9 Ansible Health, Inc., Mountain View, California, United States of America; 10 Laboratory for Computational Physiology, Massachusetts Institute of Technology, Cambridge, Massachusetts, United States of America; 11 Department of Bioinformatics, Harvard Medical School, Boston, Massachusetts, United States of America; Khalifa University of Science and Technology, UNITED ARAB EMIRATES

## Abstract

The application of machine learning and artificial intelligence to clinical settings for prevention, diagnosis, treatment, and the improvement of clinical care have been demonstrably cost-effective. However, current clinical AI (cAI) support tools are predominantly created by non-domain experts and algorithms available in the market have been criticized for the lack of transparency behind their creation. To combat these challenges, the Massachusetts Institute of Technology Critical Data (MIT-CD) consortium, an affiliation of research labs, organizations, and individuals that contribute to research in and around data that has a critical impact on human health, has iteratively developed the “Ecosystem as a Service (EaaS)” approach, providing a transparent education and accountability platform for clinical and technical experts to collaborate and advance cAI. The EaaS approach provides a range of resources, from open-source databases and specialized human resources to networking and collaborative opportunities. While mass deployment of the ecosystem still faces several hurdles, here we discuss our initial implementation efforts. We hope this will promote further exploration and expansion of the EaaS approach, while also informing or realizing policies that will accelerate multinational, multidisciplinary, and multisectoral collaborations in cAI research and development, and provide localized clinical best practices for equitable healthcare access.

## Author summary

The development of effective and scalable algorithms that are executable at the frontlines of clinical care require robust data infrastructure, data sharing and usage capabilities that do not compromise patient confidentiality, and flexibility in creating partnerships across various departments, industries, and sectors. To achieve this in a sustainable and replicable manner, the EaaS approach has three components; 1. A global coalition of AI clinicians accessible via the MIT-CD network to support the translation, customization, innovation, and external validations of algorithms, 2. Training opportunities provided via MIT-CD affiliated institutions to educate clinically informed engineers and data literate clinicians, and introduce them to open source databases, and 3. Networking opportunities and events offered by the MIT-CD consortium to share best practices and offer chances for collaboration on innovative research in multidisciplinary teams.

## 1. Introduction

Machine learning algorithms have been lauded as indispensable tools in solving complex challenges across various fields ranging from photography to self-driving vehicles, to even AI-augmented legal defense [1]. The wide-ranging applicability of these algorithms have brought about the development of services that aim to provide largely turnkey solutions for non-experts to build up their own algorithms given arbitrary datasets and labels. While the algorithms that are produced may initially seem of high quality, when put into practice they tend to suffer from significant issues such as the inability to generalize [2], systemic bias [3], and in some cases, inaccurate and non-reproducible predictions. The development of effective and scalable algorithms that are executable at the frontlines of clinical care require robust data infrastructure, data sharing and usage capabilities that do not compromise patient confidentiality, and flexibility in creating partnerships across various departments, industries, and sectors [2].

As interest in the application of machine learning in healthcare soars, similar turnkey approaches are being observed in healthcare. Not surprisingly, such efforts have translated poorly to clinical practice [2], as clinical AI (cAI) support tools are predominantly created by non-domain experts [4]. Reliable cAI requires large-scale data availability for development with subsequent local population level re-development and tuning [2,5], while ensuring clinical workflow compatibility. Yet, healthcare facilities are reluctant to openly share data and few healthcare professionals are capable of integrating data science and biomedical informatics with clinical insight [5]. Additionally, while cAI has been demonstrably cost-effective [6,7], only 4 of the 130 Food and Drug Administration (FDA) approved AI devices have been prospectively validated [8,9]. These challenges are further exacerbated when unvalidated and possibly non-generalizable algorithms are purchased by hospitals with the intention of improving clinical outcomes and resolving disparities in healthcare delivery. This not only marginalizes hospital systems that lack the resources for full-scale cAI development and procurement, but the product purchased may be unusable, with current algorithms demonstrating a lack of transparency behind their creation, and particularly with regard to any biases in the data used [10,11]. Furthermore, in the era of COVID-19, the demand for dynamic, lean, and multilateral efforts have become ever more apparent.

## 2. cAI and its current challenges

cAI is the application of machine learning and AI that assists with clinical decisions through process optimization, advancements in basic and clinical research and development, diagnostic and prognostic clinical decision support, in-hospital and extra-hospital patient support tools, and population-level applications to identify epidemics and understand non-communicable chronic diseases [12]. cAI development requires large and well-managed datasets permitting initial algorithm construction, a clinical validation feedback loop consisting of technical and clinical experts, localized application that considers the target community’s demographic and socioeconomic contexts, infrastructure for visualization deployment, and financial resources, all of which are requirements that differ based on the clinical question posed. These requirements have also locked cAI within major academic medical centers, limiting current options for cAI integration to institutions independently devoting a large sum of resources so as to build an in-house team, or to institutions opting-out of using cAI tools entirely, potentially placing one’s hospital at distinct disadvantage (Table 1).

**Table 1:** High-level overview of the current options for cAI integration at hospital level [2,13].

Option	Potential Risks/Limitations	Financial Costs	Time
**Purchase cAI aaS solutions or outsource to third party**	1. Not customizable to all settings, may require local retraining to improve efficacy 2. Few options provide holistic solutions for hospital-wide implementation 3. No in-house capacity building or method of verifying/ reproducing cAI solutions	While costs may lower as a result of better resource optimization and predictions [6,7], the aaS solution itself requires high subscription fees	Implementation may be short yet training staff to learn how to use the aaS solutions may take time
**Build an in-house cAI team** [4]	1. High cost to hire and/or train staff to create the team 2. Security risk/data breach/loss 3. Unclear return on investment	While costs may lower as a result of better resource optimization and predictions, hiring and training costs are expensive	Both implementation and training may take a substantial amount of time
**Opt out of using cAI tools**	1. Difficulty improving clinical practices for better outcomes 2. Difficulty to reduce unnecessary costs effectively 3. Widening gaps in clinical practice and overall performance with other health facilities	No costs are used for cAI tools, but potential cost-saving opportunities are lost	No time used to implement cAI tools, and little to no time saved as a result

cAI benefits from multicentered, multi- or transnational, and culturally relevant approaches, which unfortunately may be incompatible with the structure of traditional academic medical systems. Although purchasing cAI algorithms may be efficient to implement [14,15], a vast number of factors determine and influence health systems, and the creation of cAI itself without extensive validation is insufficient to spur sustainable change. Collecting data in a manner that can train cAI to suit local populations and combat bias requires considerable amounts of scrutiny and resources; further, cAI specialists are also scarce [16] and the returns on investments are yet to be determined [16,17]. In addition, comprehensive understanding of each target clinical setting requires teamwork, domain expertise, and long-term commitment. Table 2 describes the overview of cAI stakeholders.

**Table 2:** cAI stakeholder overview.

Stakeholder	Potential Challenges	Potential Strengths
**Hospital/Healthcare Organizations**	• Typically cannot afford to hire data scientists/in-house AI team or finance the data and computational infrastructure needed for analysis • Sharing data is not financially incentivized	• Has clinical data that can serve as a knowledge bank for understanding community health and local challenges
**Academia/Universities**	• Does not have access to clinicians/clinical insight	• Has resources (e.g. human resources, expertise, cAI infrastructure), to develop AI tools
**Industry**	• Does not have access to a wide variety of clinicians or health data	• Has the highest security infrastructure amongst the stakeholders that hospitals cannot afford
**Government**	• Either does not have relevant healthtech policies to direct nationwide cAI implementation or solutions to realize its policies and standardize country level cAI implementation	• Has the power and resources to implement cAI in a standardized and accelerated manner
**Coalition**	Permits all stakeholders to overcome individual pain points and fulfill needs to address and improve clinical challenges through collaborative and coordinated effort	

## 3. The “Ecosystem as a Service (EaaS)” approach

**Fig 1:**
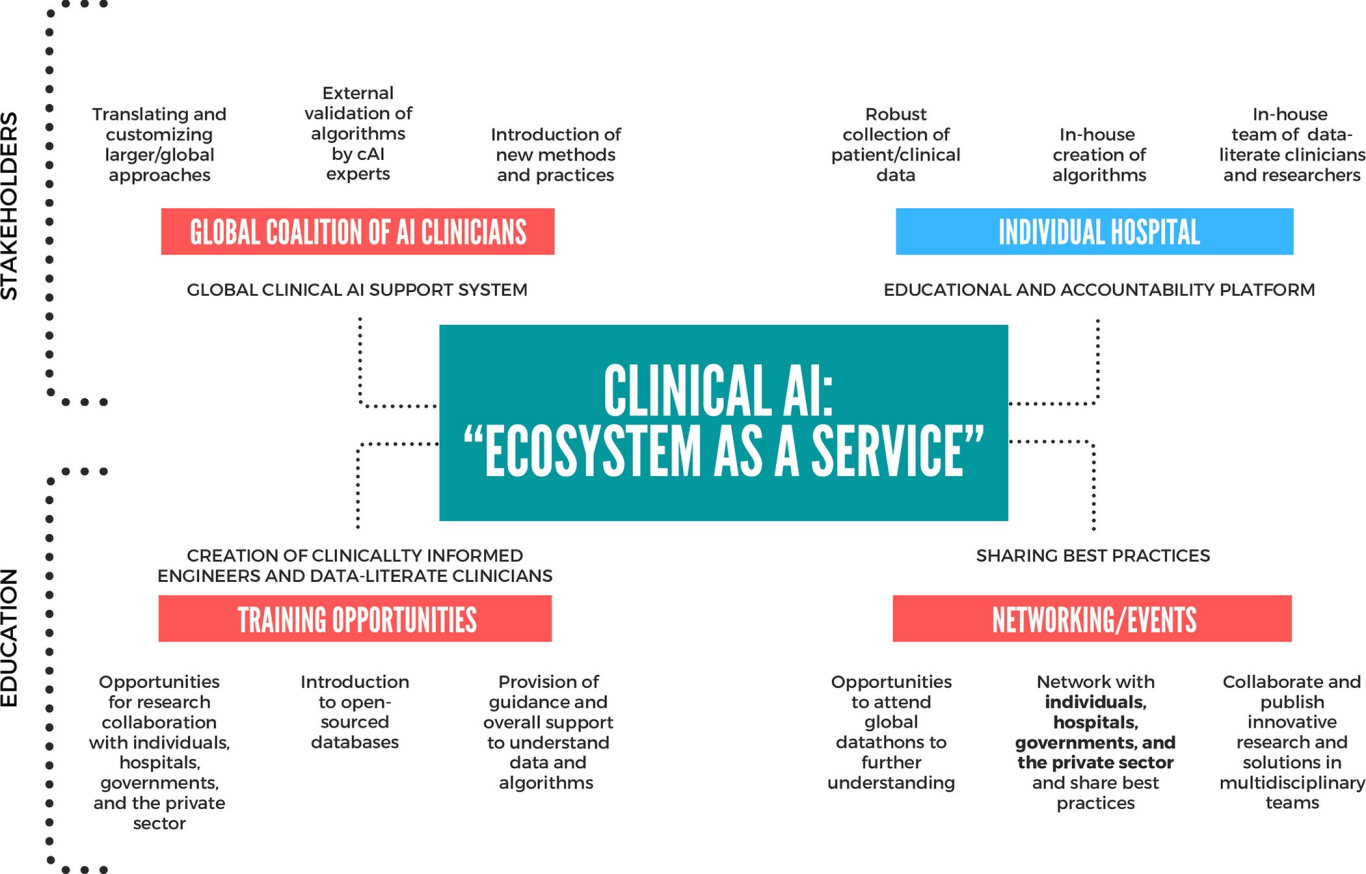
“Ecosystem as a service” for cAI. Overview of the EaaS approach for cAI with its three main components: 1. Global coalition of AI clinicians, 2. Training opportunities, and 3. Networking opportunities, integrated with an individual hospital system.

To overcome the challenges in cAI development and implementation, while leveraging the strengths of each stakeholder, the MIT Critical Data (MIT-CD) consortium developed the “Ecosystem as a Service (EaaS)” approach for sustainable and replicable cAI utilization. The MIT-CD consists of university departments, laboratories, independent academic institutions, research groups, national centers, industry representatives, and medical departments from around the globe in the field of computer science, electrical engineering, physics, or mathematics that are engaged in research in and around data that has a critical impact on human health. The EaaS approach began with the MIT Laboratory for Computational Physiology (LCP)’s efforts to use modeling, signal processing, pattern recognition, and machine learning for the development and refinement of methods to analyze data and generate predictive models for patient care. The LCP launched the Medical Information Mart for Intensive Care (MIMIC) database in the early 2000s, an open-source and free dataset of more than 60,000 de-identified intensive care unit admission records, which has since become the golden standard for public clinical databases [18,19]. The LCP network grew as interest in the MIMIC database and collaborators increased, and developed into its current MIT-CD coalition with the launch of the initial MIT Critical Data Conference in 2014 which invited academia, government, and industry leaders to discuss the pitfalls and challenges of big data in health care [20]. This also brought about the emergence of “datathons”, (a portmanteau of “data” combined with “hackathon”, indicating the application of the hackathon model to data analytics) which convenes experts from various scientific disciplines and industry involved in creating and using patient data and algorithms for collaboration, group learning, error checking, and methodological review during the initial design and subsequent phases of cAI development and research [21]. The combination of these efforts with iterative feedback and refinement with various stakeholders has shaped the current EaaS approach, which is described in Fig 1.

The EaaS approach has three components; 1. A global coalition of AI clinicians offered via the MIT-CD network to support the translation, customization, innovation, and external validations of algorithms, 2. Training opportunities provided via MIT-CD affiliated institutions to rear clinically informed engineers and data literate clinicians, and introduce them to open source databases, and 3. Networking opportunities and events offered by the MIT-CD consortium to share best practices and offer chances for collaboration on innovative research in multidisciplinary teams. Within the MIT-CD consortium, these areas are supported via grants or external sponsorships, and clinicians, engineers, and institutions are able to participate in the ecosystem and join the MIT-CD consortium at a very low cost or for free, depending on the type of partnership. The EaaS approach considers accessibility to cAI for all hospitals regardless of location and available resources and contributes to addressing and solving global health challenges. Further expansion of the ecosystem including availability of data and its sustainability is currently being discussed.

**Fig 2:**
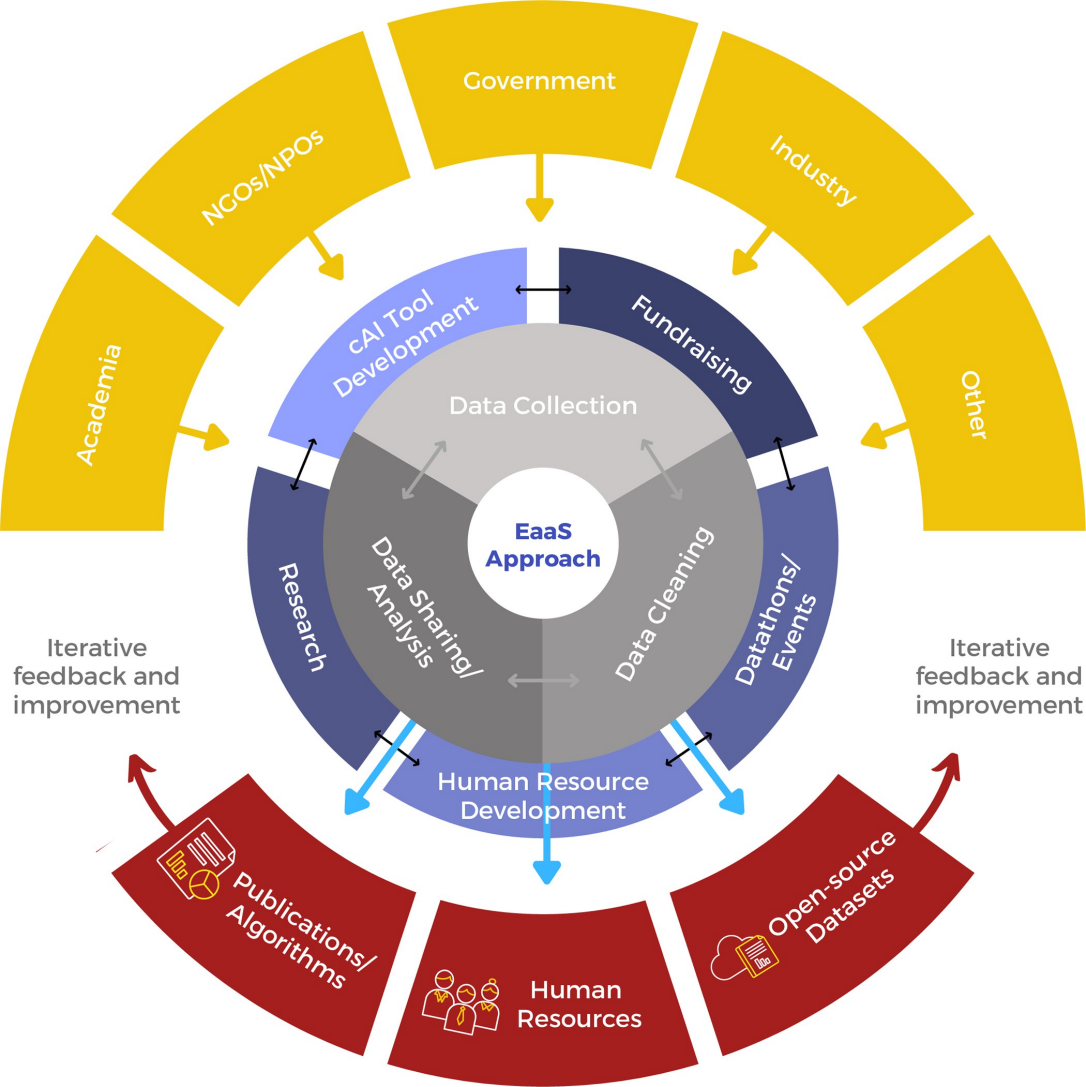
Stakeholders and Operations of the EaaS approach. High-level depiction of the stakeholders included in the EaaS approach, its operational components, as well as its iterative nature.

The MIT-CD’s EaaS approach provides a transparent educational and accountability platform for clinical and technical experts to collaborate, develop, and advance cAI. Hospital systems that monetize raw clinical data may not initially be incentivized by this approach; yet raw clinical data without curation is analogous to crude oil, which without distillation has minimal use or value. During the mid-2010s, MIT-CD and Philips recognized the importance of establishing a decentralized education platform for analyzing free and open source clinical data and have generated significant academic development and dataset analysis beyond what each group alone could achieve since. In other words, the EaaS approach permits the sharing of perspectives, experiences, and expertise, and leads to the development of new technologies that are clinically meaningful, academic publications that aid in providing evidence for unanswered clinical questions, and contribute to overall improved value-based patient care. The EaaS approach can be used for seamless and effective cAI adoption within large healthcare systems while benefiting smaller, independent, rural, and less resourced partners around the world (Fig 2).

MIT-CD’s initial implementation efforts of the EaaS approach have shown promising results in improving healthcare access and clinical care through new and refined approaches to interpreting data along with partner universities and research laboratories around the world. Since 2018, MIT-CD has held the “Big Data Machine Learning in Healthcare” datathon events in Japan, which was co-hosted with Google in 2019, bringing together over 200 individuals and a variety of key academic institutions and organizations involved in patient care and healthtech service provision [22]. These events have not only provided potential solutions to realize the Japanese government’s “Society 5.0” policy aimed to lead Japan to become the first country to achieve a human-centered society by incorporating advanced technologies in diverse industries and social activities and fostering innovation [23], but have also led to the development of national-level ICU mortality prediction models to further customized care and treatment [23,24].

In 2019, MIT-CD also held the first “Big Data Bhutan and Asia eHealth Information Network (AeHIN) Convergence Workshop on Enterprise Architecture” together with the Khesar Gyalpo University of Medical Sciences of Bhutan (KGUMSB) and the Bhutanese Ministry of Health (MoH) [25]. Bhutan is in its inaugural stages of embracing and adopting innovative technological development of AI, quantum computing, blockchain, machine learning, big data, Internet of Things (IOT), virtual reality, and augmented reality [25], especially to build capacity in order to leverage the advantages of these modern technologies for the improvement of healthcare services and other socio-economic development. Throughout the course of preparing and executing the workshop as well as planning the subsequent steps for addressing the discovered challenges, the MIT-CD ecosystem aided the rearing of human resources necessary to develop Bhutan’s health data infrastructure for the collection, storage, and maintenance of all health-related data, while facilitating networking and collaborative efforts between the Bhutanese government, academic institutions, private sector, and oversea partners. The EaaS approach is also currently being deployed in Africa and in Asia through partnerships that integrate private sector resources to enhance public sector health care access and building capacity for collaborative research and patient-centric tools.

## 4. Conclusion: Future prospects

The MIT-CD EaaS approach offers a sustainable and cost-effective option for cAI implementation and development among researchers, service providers, and consumers. Realizing the EaaS approach for mass deployment still faces several hurdles; it requires 1. the pooling of best practices across wider regions in an open, collaborative, and holistic manner, 2. a pricing structure that offers a wide variety of solutions to meet the needs of diverse healthcare systems while considering insurance and reimbursement, and 3. the accumulation, de-identification, and sharing of health data at the hospital, regional, and country level, permitting equitable access to healthcare data. The EaaS approach’s core model of interdisciplinary teamwork has thrived with the enhanced promotion of digital communication and global coordination and has been resilient even in the era of COVID-19. Although questions remain in how to translate the movement to an independent industrialized consortium, with further exploration and expansion, the EaaS approach could permit acceleration of multinational, multidisciplinary, and multisectoral collaborations in cAI research and development, providing localized clinical best practices for equitable healthcare access.
